# Hop tests and psychological PROs provide a demanding and clinician-friendly RTS assessment of patients after ACL reconstruction, a registry study

**DOI:** 10.1186/s13102-020-00182-z

**Published:** 2020-05-13

**Authors:** Ramana Piussi, Susanne Beischer, Roland Thomeé, Eric Hamrin Senorski

**Affiliations:** 1 Sportrehab Sports Medicine Clinic, Stampgatan 14, SE-411 01 Gothenburg, Sweden; 2 Unit of Physiotherapy, Department of Health and Rehabilitation, Institute of Neuroscience and Physiology, Sahlgrenska Academy, University of Gothenburg, Box 455, SE-405 30 Gothenburg, Sweden

**Keywords:** Knee, Evaluation, Test batteries, Anterior cruciate ligament

## Abstract

**Background:**

There is growing interest in assessing psychological well-being in patients after anterior cruciate ligament (ACL) reconstruction. It is unknown whether an assessment of psychological outcome in addition to tests of muscle function can facilitate decisions on return to sport (RTS). Therefore, the aim of this study was to evaluate passing rates in different physical RTS test batteries, with and without the inclusion of psychological outcome measures 1 year after ACL reconstruction.

**Method:**

In this cross-sectional cohort study a total of 320 patients (51% men) aged 18–65 years were included 1 year after ACL reconstruction**.** Passing rates on different muscle function (MF) test batteries (with results presented as Limb Symmetry Index (LSI)), consisting of knee extension and flexion strength tests, 3 hop tests, and 2 psychological patient-reported outcomes (PROs); Quality of Life subscale from the Knee injury and Osteoarthritis Outcome Score (KOOS QoL) and ACL Return to Sport after Injury (ACL-RSI), were evaluated 1 year after ACL reconstruction. Muscle function test batteries comprised: 2 MF tests (vertical hop and hop for distance; pass = 90% LSI); 2 MF tests and 2 PRO (pass = 90% LSI, 62.5 points on KOOS QoL and 76.6 points on ACL-RSI), 5 MF tests (2 strength and 3 hop tests, pass = 90% LSI), and 5 MF tests and 2 PRO (pass = 90% LSI, 62.5 points on KOOS QoL and 76.6 points on ACL-RSI).

**Results:**

Passing rates in the different test batteries were 47% for 2 MF tests, 19% for 2 MF tests and 2 PROs, 29% for 5 MF tests and 13% for 5 MF tests and 2 PROs. The use of psychological PROs together with tests of muscle function gave the lowest passing rate (13%). There was a very strong correlation between passing 2 hop tests and 2 PROs and passing 5 MF tests (rφ = 0.41) as well as passing 5 MF tests and 2 PROs (rφ = 0.79).

**Conclusion:**

The use of hop tests together with psychological PROs provides a clinician-friendly RTS test battery for assessment 1 year after ACL reconstruction as the passing rate was 19% when using 2 hop-tests combined with 2 PROs, compared with 29% when using 5 tests of MF requiring advanced testing equipment.

## Introduction

After an anterior cruciate ligament (ACL) reconstruction, up to 30% of patients suffer a second knee injury within 5 years from surgery [1]. Consequences associated with a second ACL injury are for instance lower level of physical activity, knee pain and knee-joint osteoarthritis [2, 3]. Reaching return to sport (RTS) criteria based on objective assessments of muscle function (MF) in the lower extremity, prior returning to sport, can reduce the risk of a second ACL injury [4, 5]. Since  a safe RTS after an ACL injury is a milestone for a majority of patients, it recieves much attention [6, 7]. A proper assessment of MF after ACL injury and reconstruction should comprise measures of quadriceps and hamstring strength as well as measures of functional performance, such as hop tests [8]. As an athletic injury always is followed by a psychological response, psychological outcome measures have become more common in the assessment of patients with an ACL injury [9–11].

In a recent systematic review of clinical practice guidelines (CPG) for rehabilitation after ACL reconstruction, the evaluation of psychological measures was recommended in 2 out of 5 CPGs [12]. Despite that the importance of psychological factors during rehabilitation and RTS has been widely recognized [13, 14], its implementation in the evaluation prior to RTS is scarce. Time and tests of physical performance, are the most common used RTS criteria [15].

Results from only hop performance can be insufficient as RTS criteria. Furthermore, as demands increase by adding more MF tests, the passing rate (the proportion of patients reaching a given cut-off value) is reduced [16–18]. It is, however, unknown if adding an assessment of psychological outcome to MF tests will result in different passing rates, resulting in a better foundation for decisions on RTS.

The aim of this study was therefore to evaluate passing rates in different physical RTS test batteries, with and without the inclusion of psychological outcome measures 1 year after ACL reconstruction.

## Methods

This cross-sectional study was based on data extracted from a rehabilitation outcome registry, Project ACL, on 8 February 2019. Project ACL was established in 2014 and aims to improve the care of patients with an ACL injury through the use of regular assessments as well as to provide patients and clinicians with treatment feedback. Data are collected prospectively at predefined follow-ups with ACL injury or ACL reconstruction as baseline [19–21]. The follow-up data consist of validated tests of MF and patient-reported outcomes (PROs). The patients undergo individualized rehabilitation under supervision of a registered physical therapist. Ethical approval has been obtained from the Regional Ethical Review Board (registration numbers: 265–13, T023–17).

In the present study, data from the 1-year follow-up were extracted for analysis. Patients included in the registry were eligible if: aged 18–65 years, had undergone a unilateral ACL reconstruction and attended Project ACL’s 1-year follow-up. Patients were excluded if any of the following criteria was met; registered with a second ACL injury, had not performed 1 or more of the 5 tests in the battery of MF tests, or had not responded to the Knee injury and Osteoarthritis Outcome Score, subscale Quality of Life (KOOS QoL) or the ACL Return to Sport after Injury scale (ACL-RSI).

### Muscle function

The tests of MF comprised of 2 strength and 3 hop tests. Patients are required to go through a detailed familiarization procedure with their responsible physical therapist before they are tested in Project ACL. Before testing, patients performed a standardized warm up of 10 min on a stationary bike and sub maximum trials on each test (Table 1) [22].

**Table 1:** Tests of muscle function

	Degrees of movement	Practice trials n (% of 1RM)	Test trials (n)	Rest between test trials (seconds)	Units
Knee extension	90°-0°	10 (50%);	3–4	40	Newton meters
		10 (75%);			
		1–2 (90%)			
Knee flexion	0°-90°	10 (50%);	3–4	40	Newton meters
		10 (75%);			
		1–2 (90%)			
Vertical hop	–	2	3	20	Centimeters
Hop for distance	–	2	3–5	20	Centimeters
Side hop	–	–	30 s	180	Number of hops

n = number; 1RM = one repetition maximum

Maximum concentric knee muscle strength was tested in unilateral knee extension and knee flexion at 90°/second using an isokinetic dynamometer (Biodex System 4; Biodex Medical System, Shirley, NY, USA). The Biodex dynamometer is reliable for testing muscle strength [23]. Peak torque in Newton meters (Nm) is used for analysis in this study.

Hop performance is measured with 3 single-leg hop tests: vertical hop (Muscle lab, Ergotest Technology, Oslo, Norway), hop for distance and a 30-s side-hop test. Each hop test was performed with the patients holding their hands behind their back. For the vertical hop, the time from take-off to landing was converted into hop height in centimeters. In the hop for distance test, the distance between top of the toes at take-off to heel at landing was measured in centimeters. For the 30 s side hop test, one trial per leg was allowed, where the patient was instructed to hop as many times as possible over 2 lines 40 cm apart. The number of hops was recorded. The hop tests have good validity and reliability for measuring hop performance in patients with an ACL injury or reconstruction [22].

The results of the tests are presented as the Limb Symmetry Index (LSI), which is the result for the injured leg, divided by the result for the uninjured leg, multiplied by 100 and expressed as a percentage.

### Psychological patient-reported outcome

The KOOS is valid and reliable for patients with an ACL injury [24]. The KOOS comprises 5 subscales: Pain, Symptoms, Activity of daily living, Function in sports and recreation, and QoL. Each item is rated from 0 to 4 on a 5-point Likert scale. In this study, the subscale of QoL was used.

The ACL-RSI has been developed to measure an athlete’s psychological readiness to return to sport. The ACL-RSI is reliable, valid, and widely used to predict return to sport [25, 26]. Each item is graded from 0 to 10, where 10 indicates the greatest readiness to return to sport. In this study, the 12-item version was used [26].

The Tegner Activity Scale (Tegner) is meant to reflect how strenuous a physical activity is for the knee [27]. The scale ranges from 0 to 10, where 10 indicates the most knee strenuous physical activity. The scale has good validity for patients with an ACL reconstruction [28]. In the present study, a modified version was used [20]. The modified version does not contain any “0” value, which represents “sick leave or disability pension because of knee problems” in the original version of the Tegner, and has recreation sports as a choice up to level 9.

The PROs were chosen as the ACL-RSI is specifically developed for patients with ACL injuries, and has been reported with the highest methodological quality to assess patients with ACL reconstruction [29]. The QoL is a subscale of the KOOS which reflects the impact of the knee injury on patient’s life and is commonly used to assess patients after primary ACL injury [30].

### Test batteries

In this study, 4 different test batteries were evaluated. The names of the test batteries subsequently used in this paper are presented in Table 2.

**Table 2:** Test batteries used in the present study

Type of test	Strength tests	Hop tests	PROs
2 MF tests		• vertical hop	
		• hop for distance	
2 MF tests and 2 PROs		• vertical hop	• KOOS QoL
		• hop for distance	• ACL-RSI
5 MF tests	• knee extension	• vertical hop	
	• knee flexion	• hop for distance	
		• side hop	
5 MF tests and 2 PROs	• knee extension	• vertical hop	• KOOS QoL
	• knee flexion	• hop for distance	• ACL-RSI
		• side hop	

ACL-RSI = Anterior Cruciate Ligament Return to Sport after Injury; KOOS QoL = Knee injury and Osteoarthritis Outcome Score, Quality of Life subscale; MF = Muscle Function; PROs = patient-reported outcomes

For the 2 MF tests, the vertical hop and the hop for distance were chosen as Abrahams et al. [31] reported these tests as the most commonly used functional tests following ACL reconstruction. Furthermore, the 2 hop tests require minimal equipment, cost or training compared to isokinetic testing and were chosen as clinician friendly. The battery of 5 MF tests was chosen as current consensus criteria for assessment of patients after ACL reconstruction include testing of both muscle strength and hop performance [8].

#### Definition of passing

Passing the tests of MF was defined as achieving an LSI value of ≥90% [8]. When 2 or 5 tests of MF were taken into account, passing was achieved when the LSI was ≥90% in all tests taken into account.

For the psychological PROs, Muller et al. [32] suggested a score of 62.5 points for the KOOS QoL as a threshold for the state of “feeling well”. With regard to the ACL-RSI, McPherson et al. [33] presented that a cut-off of 76.6 points in young patients had maximal sensitivity (78%, with 39% specificity) for discriminating between patients who sustain a second ACL injury and patients who do not within 2 years from the index ACL reconstruction [34]. These 2 cut-offs for the KOOS QoL and the ACL-RSI were applied in this study and scores above the cut-offs were considered as passing.

### Statistics

Statistical analysis was performed with the Statistical Package for Social Sciences (SPSS) (version 24, SPSS Inc., Chicago, IL, USA). Mean values, standard deviations, counts and percentages were calculated and presented for demographic data. To compare passing rates between the different test batteries, the sign test was used*.* Alpha was set at < 0.05. To test correlations, the Phi coefficient was used for binary variables. Reference values used for the Phi coefficient were: > 0.05 = weak; > 0.10 = moderate; > 0.15 = strong; > 0.25 = very strong [35].

## Results

**Fig. 1:**
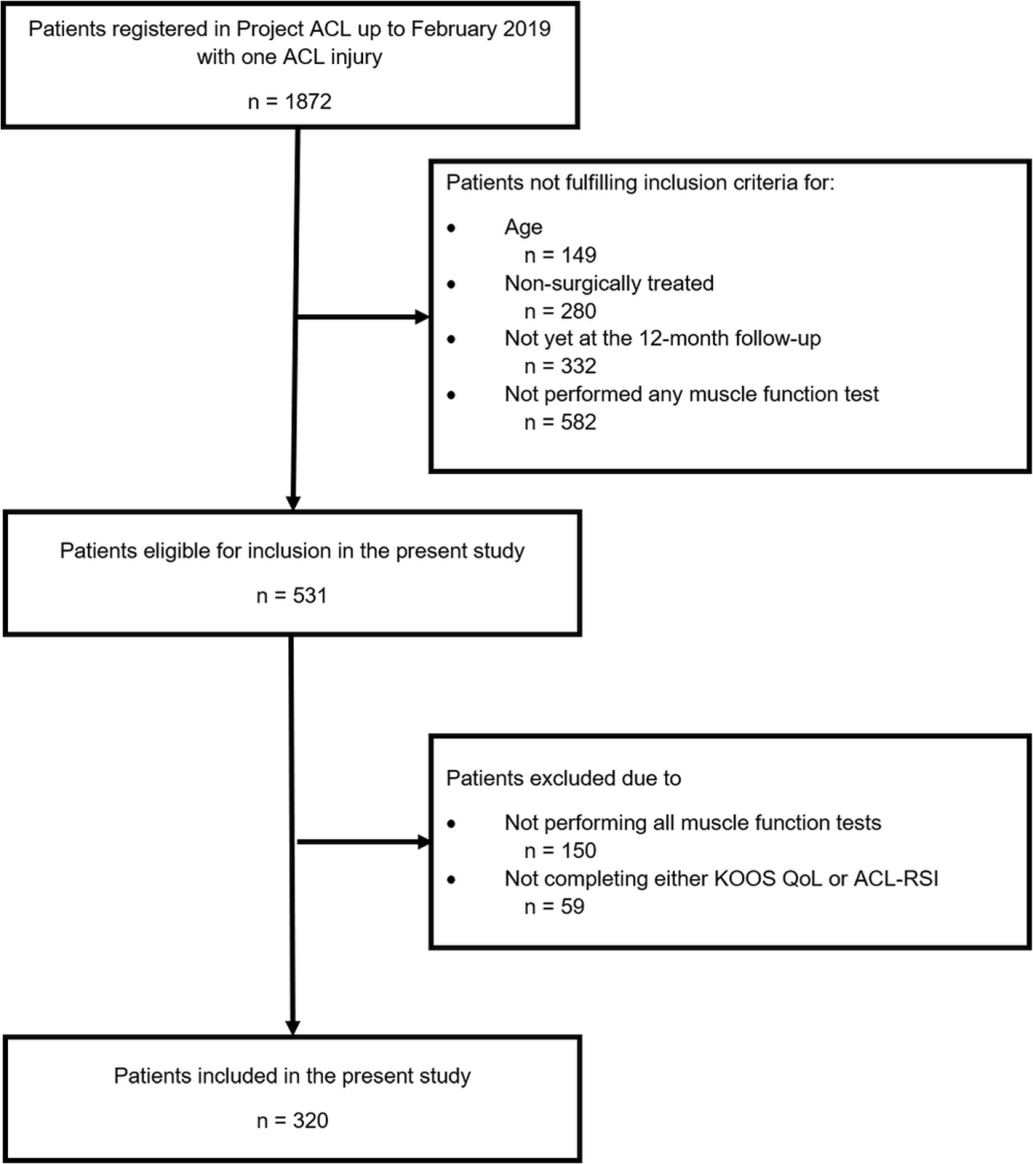
Flowchart of included and excluded patients. n = number

A total of 320 patients (51% men) met the final inclusion criteria for the study (Fig. 1).

On average, patients were 27.1 ± 9 years old and had a BMI of 23.7 ± 2 kg/m^2^ at the time of ACL reconstruction. The majority of patients underwent ACL reconstruction with a hamstring tendon autograft (85%) (Table 3).

**Table 3:** Demographic data. Mean values, standard deviations (SD), count (n) and proportions (%)

	All subjects (*n* = 320)	Men (*n* = 164)	Women (*n* = 156)
Age at reconstruction (years)	27.1 (9.4)	27.5 (8.9)	26.6 (9.9)
Height (cm)	174.7 (9)	181.5 (7)	167.5 (6)
Weight (kg)	72.8 (12)	80.8 (10)	64.3 (8)
BMI	23.7 (2)	24.5 (2)	22.8 (2)
Days between injury and reconstruction	401 (881)	439 (888)	363 (875)
Hamstring graft, n (%)	272 (85%)	139 (85%)	133 (85%)
Patellar graft, n (%)	41 (13%)	22 (13%)	19 (12.2%)
Other graft, n (%)	6 (2%)	2 (1%)	4 (2%)
Preinjury Tegner: levels, n (%)	1–5: 55 (17%)	1–5: 23 (14%)	1–5: 32 (20%)
	6–8: 151 (47%)	6–8: 71 (43%)	6–8: 80 (51%)
	9–10: 114 (36%)	9–10: 70 (43%)	9–10: 44 (28%)
1-year Tegner: levels, n (%)	1–5: 123 (38.5%)	1–5: 67 (40.8%)	1–5: 56 (35.9%)
	6–8: 127 (39.8%)	6–8: 55 (33.6%)	6–8: 72 (46.2%)
	9–10: 45 (14.1%)	9–10: 30 (18.2%)	9–10: 15 (9.6%)
	Missing: 25 (7.8%)	Missing: 12 (7.3%)	Missing: 13 (8.3%)

BMI = Body Mass Index; cm = centimeters; kg = kilograms; n = number; Tegner = Tegner Activity Scale

**Fig. 2:**
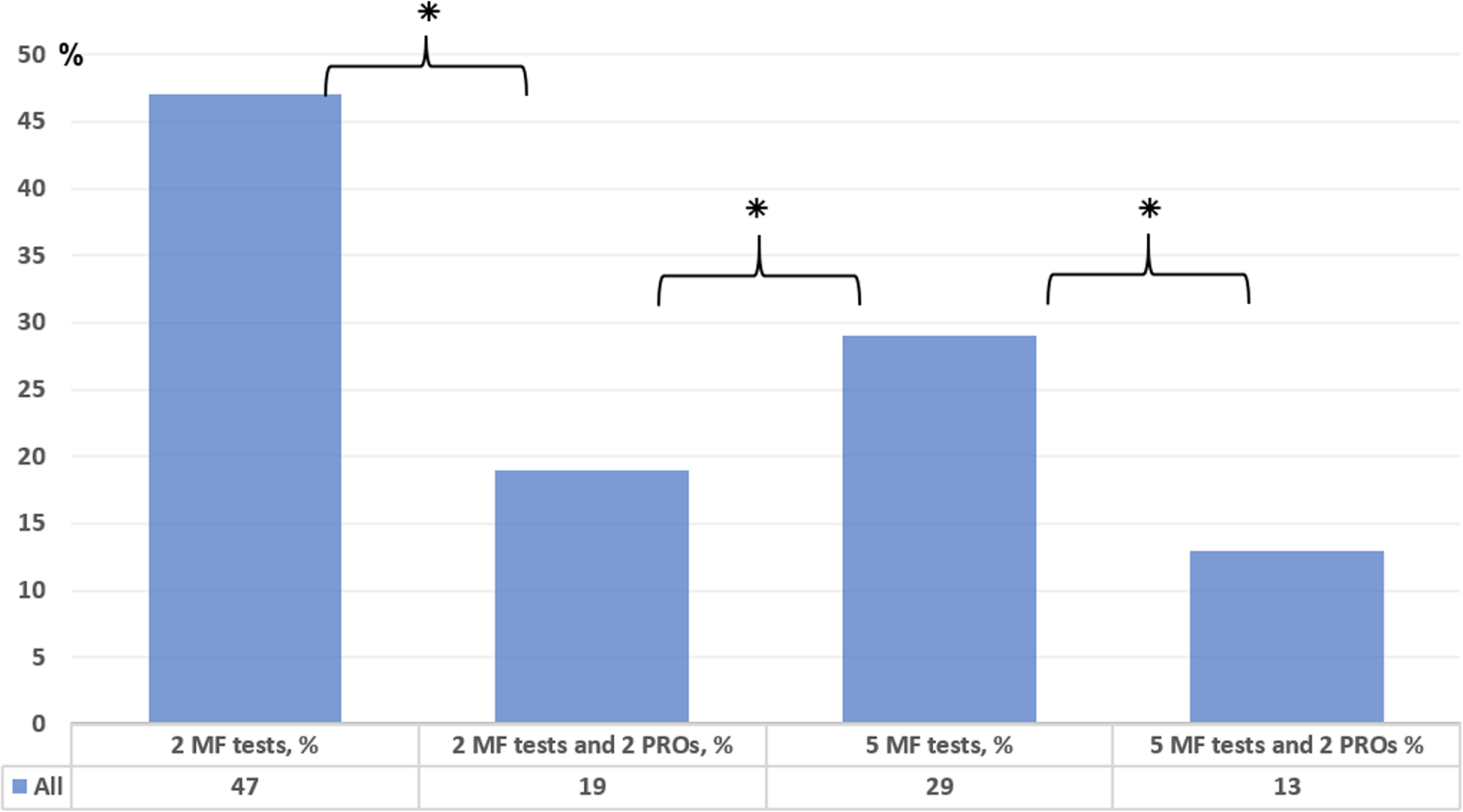
Proportion (%) of 320 patients passing the different return to sport test batteries; 2 MF = vertical hop and hop for distance; 2 MF + 2PROs = vertical hop, hop for distance, KOOS QoL and ACL-RSI; 5 MF tests = knee extension, knee flexion, vertical hop, hop for distance and side hop; 5 MF tests + 2 PROs = knee extension, knee flexion, vertical hop, hop for distance, side hop, KOOS QoL and ACL-RSI; ⁕ = *p* < 0.001; ACL-RSI = The Anterior Cruciate Ligament Return to Sport after Injury; KOOS QoL = The Knee injury and Osteoarthritis Outcome Score, subscale Quality of Life; MF = muscle function; PRO = Patient Reported Outcome

There were 47% (*n* = 152) of the patients passing *2 MF tests* (LSI ≥90%), compared with 19% (*n* = 61) passing *2 MF tests and 2 PROs* (*p* ≤ 0.001). There were 29% (*n* = 92) passing *5 MF tests*, compared with 13% (*n* = 41) passing *5 MF tests and 2 PROs* (*p* ≤ 0.001) (Fig. 2). Passing rates *(19%)* on *2 MF tests and 2 PROs* were significantly (*p* ≤ 0.001) lower than passing rates *(*29%) on *5 MF tests.*

**Fig. 3:**
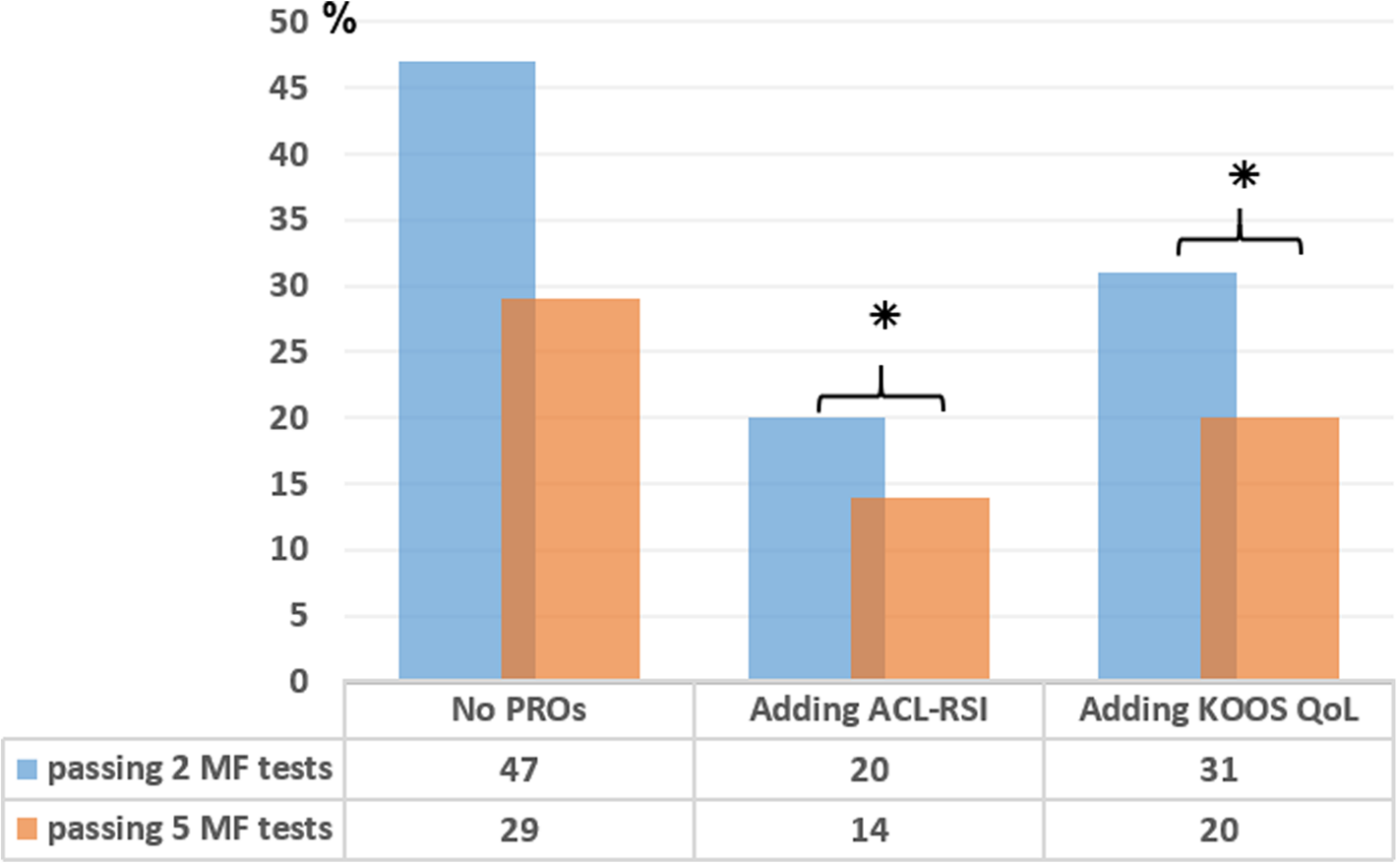
Proportion of patients passing cut-offs when adding one PRO at a time. ⁕ = p < 0.001 in comparison between passing rates when adding each of the PROs; 2 MF = vertical hop and hop for distance; 5 MF tests = knee extension, knee flexion, vertical hop, hop for distance and side hop; ACL-RSI = The Anterior Cruciate Ligament Return to Sport after Injury; KOOS QoL = The Knee injury and Osteoarthritis Outcome Score, subscale Quality of Life; MF = muscle function; PRO = Patient Reported Outcome

A total of 47% (*n* = 152) of the patients met the cut-off for ACL-RSI, while 62% (*n* = 198) met the cut-off for KOOS QoL (*p* ≤ 0.001). When adding ACL-RSI or KOOS QoL to 2 MF tests, the passing rates decreased from 47% (n = 152) to 20% (*n* = 65) (ACL-RSI) and 31% (*n* = 98) (KOOS QoL), respectively (p ≤ 0.001). When adding ACL-RSI or KOOS QoL to 5 MF tests, the passing rates decreased from 29% (*n* = 92) to 14% (*n* = 44) (ACL-RSI) and 20% (*n* = 63) (KOOS QoL), respectively (*p* ≤ 0.001) (Fig. 3).

There was no significant correlation between passing cut-offs in both PROs and passing *2 MF tests* or *5 MF tests*, respectively. Passing 2 MF tests and 2 PROs resulted in a very strong correlation with passing 5 MF tests (r_φ_ = 0.41, p ≤ 0.001) as well as passing 5 MF tests and 2 PROs (r_φ_ = 0.79, p ≤ 0.001) (Table 4).

**Table 4:** Correlation between different return to sport test batteries

	Pass both PROs	*P* values
Pass 2 MF tests	r_φ_ = −0.22	0.699
Pass 5 MF tests	r_φ_ = 0.43	0.444
	Pass 2 MF tests and 2 PROs	
Pass 5 MF tests	r_φ_ = 0.41	≤0.001
Pass 5 MF tests and 2 PROs	r_φ_ = 0.79	≤0.001

PROs, patient-reported outcomes

r_φ_ = Phi coefficient: > 0.05 = weak; > 0.10 = moderate; > 0.15 = strong; > 0.25 = very strong

## Discussion

The main finding of this study was that using an RTS test battery comprising *2 MF tests and 2 PROs* reduced the passing rate, compared with using a battery of *5 MF tests* (19% versus 29%). Interestingly, there was a very strong correlation [35] between the two different test batteries. Therefore, a clinic without advanced testing equipment to measure strength can use *2 hop tests and 2 psychological PROs* as criteria for RTS. The passing rates are comparable or even lower than the passing rates of a comprehensive battery of 5 MF tests (strength and hop). There was no significant correlation between passing MF test batteries and reaching cut-offs for either KOOS QoL or ACL-RSI, which indicates that the use of only MF tests or only psychological outcomes is likely insufficient as RTS criteria.

Our results suggest that 1 in every 2 patients passed the RTS criteria and achieved symmetrical knee function, when the decision was based on reaching leg symmetry in 2 unilateral hop tests. With test batteries that comprise more tests, the passing rates decreased, in agreement with the literature [16–18]. More tests, thus, increase the demands on the patient’s recovery after ACL reconstruction. When 5 MF tests with or without 2 PROs were used, the passing rate, compared with only 2 hop tests, the passing rates decreased from 47% to approximately 13% and 29%, respectively. The use of only 2 hop tests to determine symmetrical muscle function can, therefore, not be recommended, as approximately 30% of patients run the risk of being classified as false positives.

Current recommendations for RTS evaluation are strongly supported by results from the present study, suggesting that batteries of tests should comprise strength and hop tests, as well as PROs [36, 37]. In our cohort, a very small proportion of patients met our recommended RTS criteria at 1 year after ACL reconstruction. This result indicates that clinical settings, included in Project ACL, and responsible medical professionals for the treatment of the patients in this study, need to better prepare patients in order to make a safe RTS, i.e. increase the use of evidence-based evaluation to guide rehabilitation protocols.

The results of low psychological readiness to RTS and unacceptable low knee-related QoL suggest that some patients have recovered MF without recovering psychological impairments. Psychological factors are important during rehabilitation [38, 39], where for example, high fear of re-injury can prevent patients from returning to their preinjury level of sport [40–42]. Furthermore, a lower psychological readiness to RTS 1 year after ACL reconstruction is associated with a higher risk of a second ACL injury [34], supporting that it is important to include psychological PROs in RTS decision-making, alongside tests of MF.

In this study, a smaller proportion of patients met the criteria for an acceptable ACL-RSI compared with KOOS QoL. The ACL-RSI was developed to assess psychological readiness to RTS [25]. However, the impact returning to sport has on ACL-RSI, i.e. whether RTS leads to high psychological readiness or whether high psychological readiness leads to RTS, is yet to be studied. Patients who do not RTS after ACL reconstruction can report poor knee-related QoL up to 20 years after surgery, compared with patients who RTS [43]. However, the use of both the KOOS QoL and the ACL-RSI in this study, led to more patients being identified as not “recovered” compared with using only MF tests. Future studies are needed to better understand how individual psychological profiles are related to a safe RTS.

Given the high rate of new knee-related injuries in patients after ACL reconstruction [1, 44] and the assumption that patients who RTS might not have been ready for it, more emphasis should be placed on preparing patients for RTS test battery criteria during rehabilitation, especially as passing RTS test batteries can reduce the risk of re-injury [5].

### Limitations and strengths

A limitation of this study is that we did not determine the different test batteries effectiveness to reduce the risk of a second ACL injury. Even though there is evidence [5, 45] suggesting that patients who meet certain cut-offs in RTS test batteries have lower risk for a second ACL injury, there is an ongoing debate [46, 47] about the evidence and the validity of RTS testing.

The use of the LSI is a limitation since the patients’ healthy limb can have reduced strength after ACL reconstruction [48], meaning that tests of muscle function may overestimate the function of the operated limb [49]. Results in the present study might therefore be falsely high, which strengthens the recommendation that RTS criteria are important to meet before RTS.

Patients who suffered a second ACL rupture were excluded from the present study in order to create a group of patients that was as homogeneous as possible. Future studies will show how the different batteries of tests assessed in this study affect the risk of a new ACL injury.

In this study, the primary outcomes were results from muscle function tests and PROs. Cases where patients were unable to RTS due to unresolved impairments to the somatosensory system could therefore not be studied.

The primary strength of this study is the relatively large number of patients included. Another strength is the choice of PROs, since the KOOS and the ACL-RSI was used, and these have high methodological quality [29].

## Conclusion

The use of hop tests together with psychological PROs provides a clinician-friendly RTS test battery for assessment 1 year after ACL reconstruction as the passing rate was 19% when using 2 hop-tests combined with 2 PROs, compared with 29% when using 5 tests of MF requiring advanced testing equipment.

## Data Availability

The dataset used and/or analyzed during the current study are available from the corresponding author on reasonable request.

## Abbreviations

RTS: Return to Sport
ACL: Anterior Cruciate Ligament
MF: Muscle Function
PROs: Patient Reported Outcomes
KOOS QoL: Knee injury and Osteoarthritis Outcome Score, subscale Quality of Life
ACL-RSI: ACL Return to Sport after Injury scale
LSI: Limb Symmetry Index
SPSS: Statistical Package for Social Sciences
